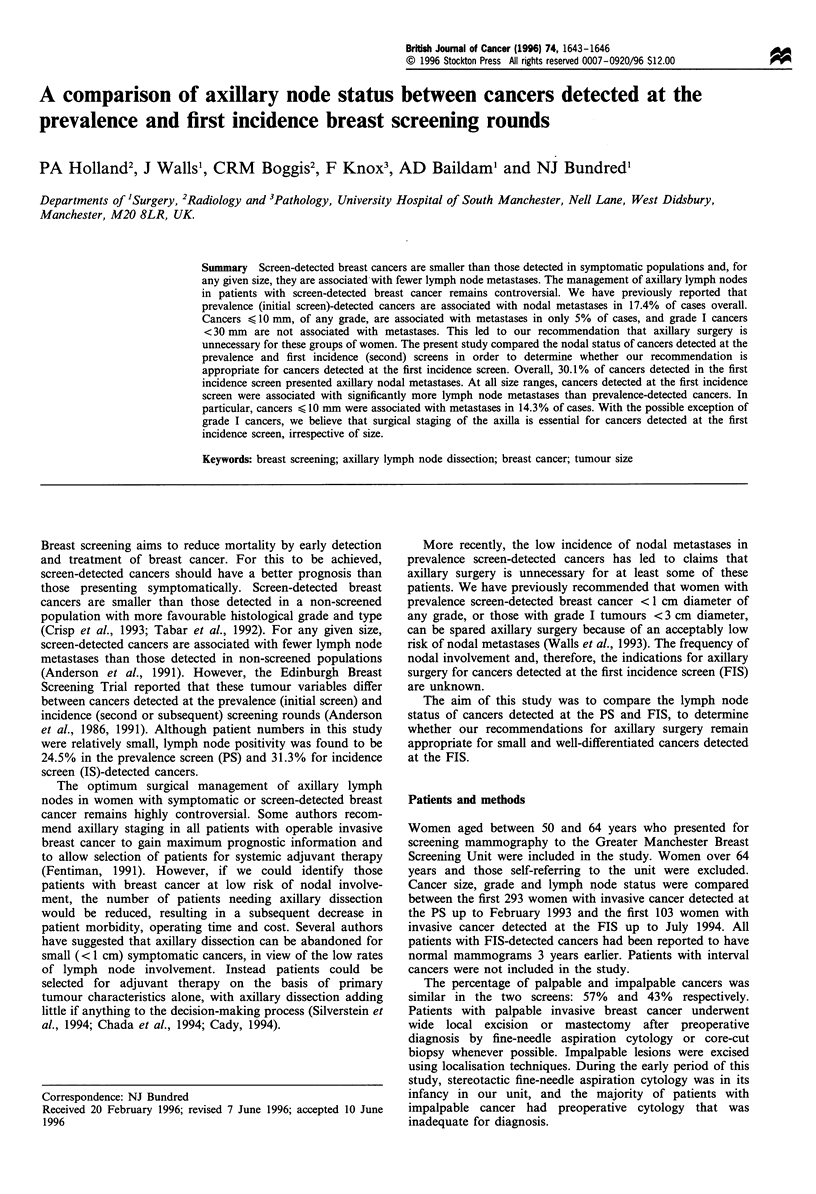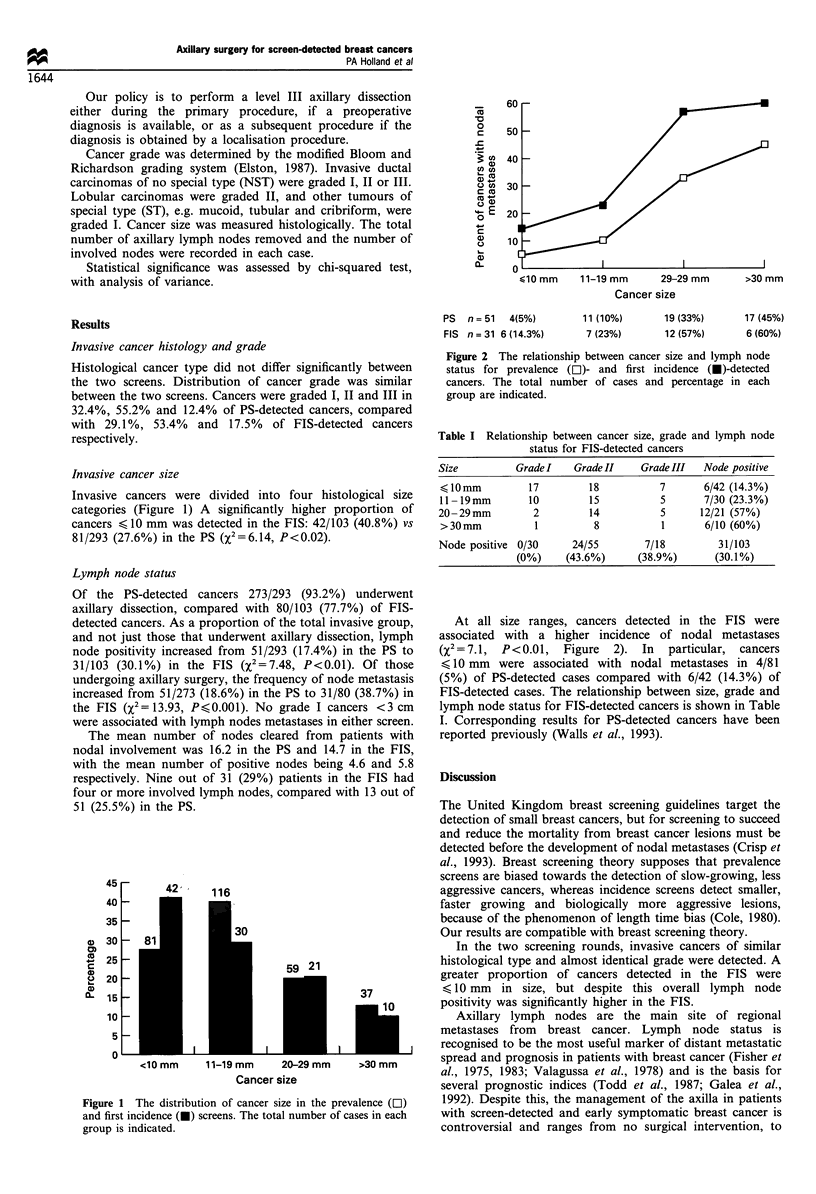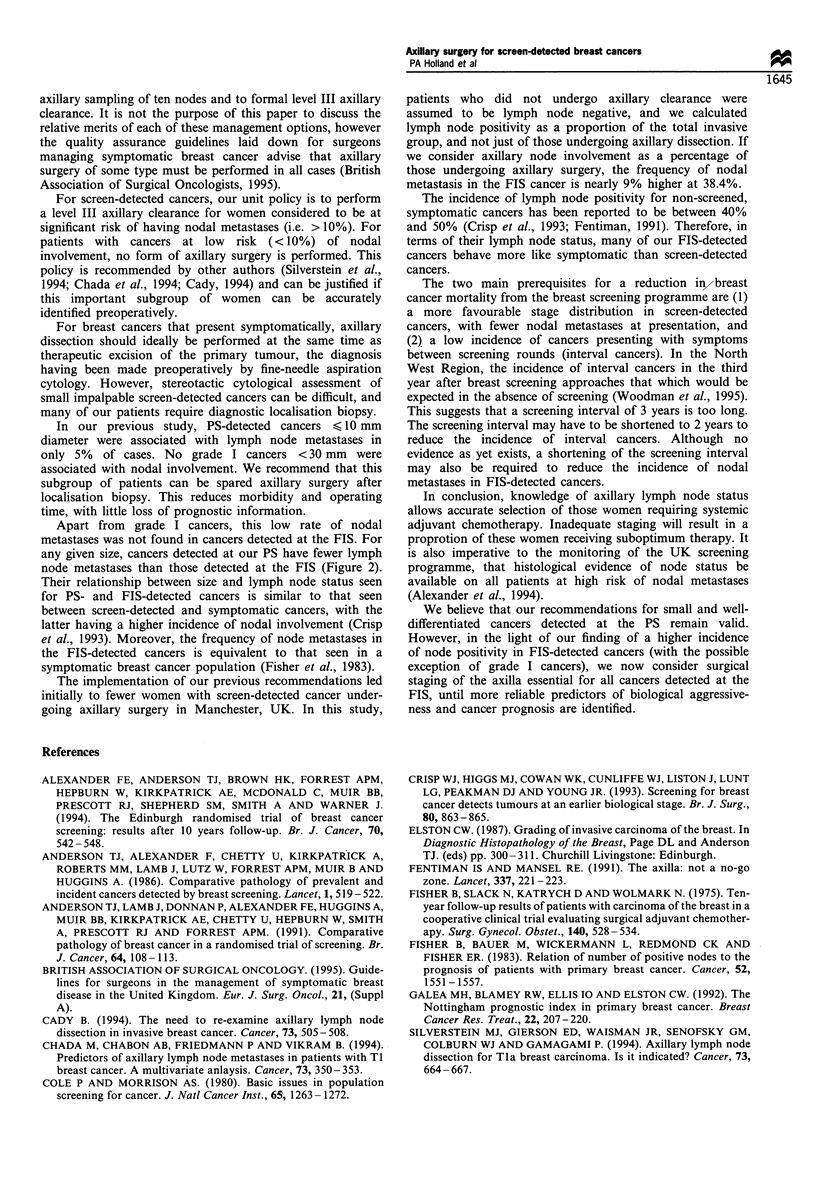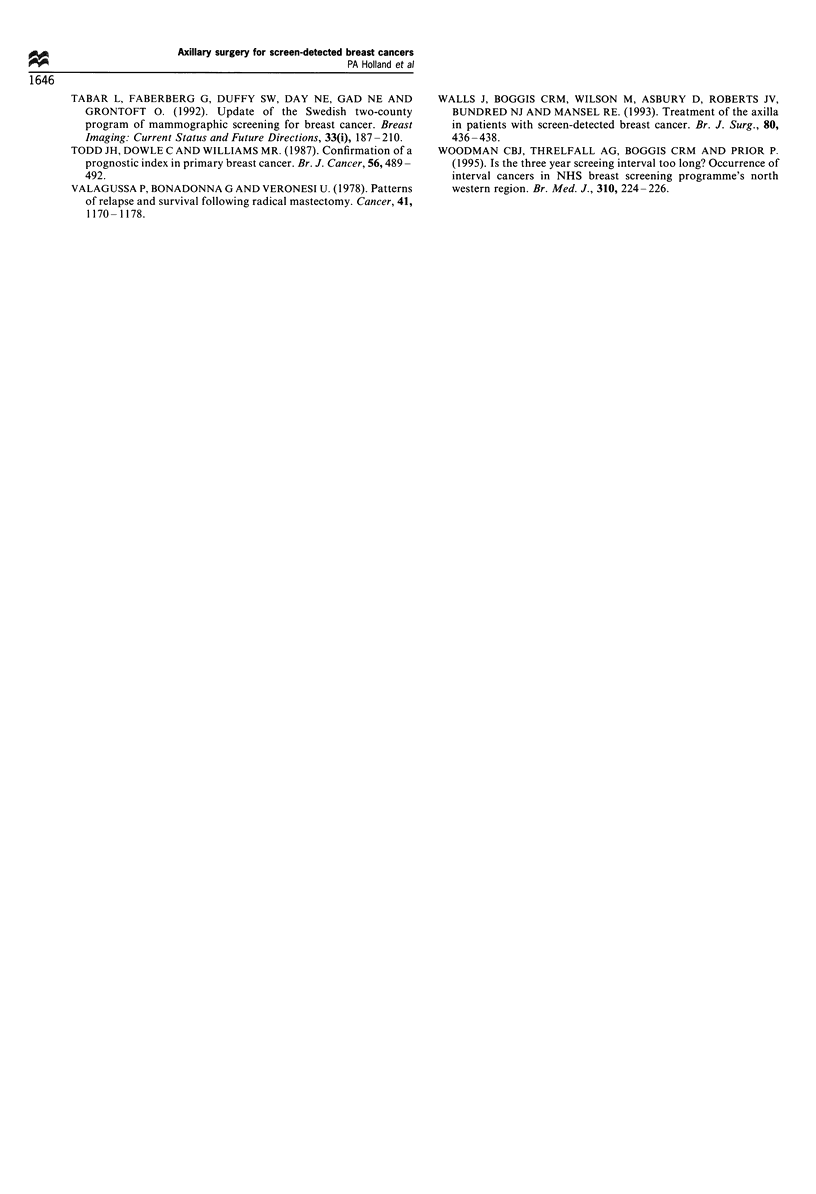# A comparison of axillary node status between cancers detected at the prevalence and first incidence breast screening rounds.

**DOI:** 10.1038/bjc.1996.602

**Published:** 1996-11

**Authors:** P. A. Holland, J. Walls, C. R. Boggis, F. Knox, A. D. Baildam, N. J. Bundred

**Affiliations:** Department of Radiology, University Hospital of South Manchester, UK.

## Abstract

Screen-detected breast cancers are smaller than those detected in symptomatic populations and, for any given size, they are associated with fewer lymph node metastases. The management of axillary lymph nodes in patients with screen-detected breast cancer remains controversial. We have previously reported that prevalence (initial screen)-detected cancers are associated with nodal metastases in 17.4% of cases overall. Cancers < or = 10 mm, of any grade, are associated with metastases in only 5% of cases, and grade I cancers <30 mm are not associated with metastases. This led to our recommendation that axillary surgery is unnecessary for these groups of women. The present study compared the nodal status of cancers detected at the prevalence and first incidence (second) screens in order to determine whether our recommendation is appropriate for cancers detected at the first incidence screen. Overall, 30.1% of cancers detected in the first incidence screen presented axillary nodal metastases. At all size ranges, cancers detected at the first incidence screen were associated with significantly more lymph node metastases than prevalence-detected cancers. In particular, cancers < or = 10 mm were associated with metastases in 14.3% of cases. With the possible exception of grade I cancers, we believe that surgical staging of the axilla is essential for cancers detected at the first incidence screen, irrespective of size.


British Journal of Cancer (1996) 74, 1643-1646
© 1996 Stockton Press All rights reserved 0007-0920/96 $12.00

### A comparison of axillary node status between cancers detected at the prevalence and first incidence breast screening rounds

PA Holland[2], J Walls[1], CRM Boggis[2], F Knox[3], AD Baildam[1] and NJ Bundred[1]

*Departments of [1]Surgery, [2]Radiology and [3]Pathology, University Hospital of South Manchester, Nell Lane, West Didsbury, Manchester, M20 8LR, UK.*

**Summary** Screen-detected breast cancers are smaller than those detected in symptomatic populations and, for any given size, they are associated with fewer lymph node metastases. The management of axillary lymph nodes in patients with screen-detected breast cancer remains controversial. We have previously reported that prevalence (initial screen)-detected cancers are associated with nodal metastases in 17.4% of cases overall. Cancers ⩽10 mm, of any grade, are associated with metastases in only 5% of cases, and grade I cancers <30 mm are not associated with metastases. This led to our recommendation that axillary surgery is unnecessary for these groups of women. The present study compared the nodal status of cancers detected at the prevalence and first incidence (second) screens in order to determine whether our recommendation is appropriate for cancers detected at the first incidence screen. Overall, 30.1% of cancers detected in the first incidence screen presented axillary nodal metastases. At all size ranges, cancers detected at the first incidence screen were associated with significantly more lymph node metastases than prevalence-detected cancers. In particular, cancers ⩽10 mm were associated with metastases in 14.3% of cases. With the possible exception of grade I cancers, we believe that surgical staging of the axilla is essential for cancers detected at the first incidence screen, irrespective of size.

**Keywords:** breast screening; axillary lymph node dissection; breast cancer; tumour size

Breast screening aims to reduce mortality by early detection and treatment of breast cancer. For this to be achieved, screen-detected cancers should have a better prognosis than those presenting symptomatically. Screen-detected breast cancers are smaller than those detected in a non-screened population with more favourable histological grade and type (Crisp *et al.*, 1993; Tabar *et al.*, 1992). For any given size, screen-detected cancers are associated with fewer lymph node metastases than those detected in non-screened populations (Anderson *et al.*, 1991). However, the Edinburgh Breast Screening Trial reported that these tumour variables differ between cancers detected at the prevalence (initial screen) and incidence (second or subsequent) screening rounds (Anderson *et al.*, 1986, 1991). Although patient numbers in this study were relatively small, lymph node positivity was found to be 24.5% in the prevalence screen (PS) and 31.3% for incidence screen (IS)-detected cancers.

The optimum surgical management of axillary lymph nodes in women with symptomatic or screen-detected breast cancer remains highly controversial. Some authors recommend axillary staging in all patients with operable invasive breast cancer to gain maximum prognostic information and to allow selection of patients for systemic adjuvant therapy (Fentiman, 1991). However, if we could identify those patients with breast cancer at low risk of nodal involvement, the number of patients needing axillary dissection would be reduced, resulting in a subsequent decrease in patient morbidity, operating time and cost. Several authors have suggested that axillary dissection can be abandoned for small (<1 cm) symptomatic cancers, in view of the low rates of lymph node involvement. Instead patients could be selected for adjuvant therapy on the basis of primary tumour characteristics alone, with axillary dissection adding little if anything to the decision-making process (Silverstein *et al.*, 1994; Chada *et al.*, 1994; Cady, 1994).

Correspondence: NJ Bundred
Received 20 February 1996; revised 7 June 1996; accepted 10 June 1996

More recently, the low incidence of nodal metastases in prevalence screen-detected cancers has led to claims that axillary surgery is unnecessary for at least some of these patients. We have previously recommended that women with prevalence screen-detected breast cancer <1 cm diameter of any grade, or those with grade I tumours <3 cm diameter, can be spared axillary surgery because of an acceptably low risk of nodal metastases (Walls *et al.*, 1993). The frequency of nodal involvement and, therefore, the indications for axillary surgery for cancers detected at the first incidence screen (FIS) are unknown.

The aim of this study was to compare the lymph node status of cancers detected at the PS and FIS, to determine whether our recommendations for axillary surgery remain appropriate for small and well-differentiated cancers detected at the FIS.

### Patients and methods

Women aged between 50 and 64 years who presented for screening mammography to the Greater Manchester Breast Screening Unit were included in the study. Women over 64 years and those self-referring to the unit were excluded. Cancer size, grade and lymph node status were compared between the first 293 women with invasive cancer detected at the PS up to February 1993 and the first 103 women with invasive cancer detected at the FIS up to July 1994. All patients with FIS-detected cancers had been reported to have normal mammograms 3 years earlier. Patients with interval cancers were not included in the study.

The percentage of palpable and impalpable cancers was similar in the two screens: 57% and 43% respectively. Patients with palpable invasive breast cancer underwent wide local excision or mastectomy after preoperative diagnosis by fine-needle aspiration cytology or core-cut biopsy whenever possible. Impalpable lesions were excised using localisation techniques. During the early period of this study, stereotactic fine-needle aspiration cytology was in its infancy in our unit, and the majority of patients with impalpable cancer had preoperative cytology that was inadequate for diagnosis.

Our policy is to perform a level III axillary dissection either during the primary procedure, if a preoperative diagnosis is available, or as a subsequent procedure if the diagnosis is obtained by a localisation procedure.

Cancer grade was determined by the modified Bloom and Richardson grading system (Elston, 1987). Invasive ductal carcinomas of no special type (NST) were graded I, II or III. Lobular carcinomas were graded II, and other tumours of special type (ST), e.g. mucoid, tubular and cribriform, were graded I. Cancer size was measured histologically. The total number of axillary lymph nodes removed and the number of involved nodes were recorded in each case.

Statistical significance was assessed by chi-squared test, with analysis of variance.

### Results

*Invasive cancer histology and grade*

Histological cancer type did not differ significantly between the two screens. Distribution of cancer grade was similar between the two screens. Cancers were graded I, II and III in 32.4%, 55.2% and 12.4% of PS-detected cancers, compared with 29.1%, 53.4% and 17.5% of FIS-detected cancers respectively.

*Invasive cancer size*

Invasive cancers were divided into four histological size categories (Figure 1) A significantly higher proportion of cancers ⩽10 mm was detected in the FIS: 42/103 (40.8%) *vs* 81/293 (27.6%) in the PS ($\chi^2=6.14$, $P<0.02$).

*Lymph node status*

Of the PS-detected cancers 273/293 (93.2%) underwent axillary dissection, compared with 80/103 (77.7%) of FIS-detected cancers. As a proportion of the total invasive group, and not just those that underwent axillary dissection, lymph node positivity increased from 51/293 (17.4%) in the PS to 31/103 (30.1%) in the FIS ($\chi^2=7.48$, $P<0.01$). Of those undergoing axillary surgery, the frequency of node metastasis increased from 51/273 (18.6%) in the PS to 31/80 (38.7%) in the FIS ($\chi^2=13.93$, $P\leqslant0.001$). No grade I cancers <3 cm were associated with lymph nodes metastases in either screen.

The mean number of nodes cleared from patients with nodal involvement was 16.2 in the PS and 14.7 in the FIS, with the mean number of positive nodes being 4.6 and 5.8 respectively. Nine out of 31 (29%) patients in the FIS had four or more involved lymph nodes, compared with 13 out of 51 (25.5%) in the PS.

**Figure 1** The distribution of cancer size in the prevalence (□) and first incidence (■) screens. The total number of cases in each group is indicated.

| | ⩽10 mm | 11–19 mm | 29–29 mm | >30 mm |
|---|---|---|---|---|
| PS $n=51$ | 4(5%) | 11 (10%) | 19 (33%) | 17 (45%) |
| FIS $n=31$ | 6 (14.3%) | 7 (23%) | 12 (57%) | 6 (60%) |

**Figure 2** The relationship between cancer size and lymph node status for prevalence (□)- and first incidence (■)-detected cancers. The total number of cases and percentage in each group are indicated.

**Table I** Relationship between cancer size, grade and lymph node status for FIS-detected cancers

| *Size* | *Grade I* | *Grade II* | *Grade III* | *Node positive* |
|---|---|---|---|---|
| ⩽10 mm | 17 | 18 | 7 | 6/42 (14.3%) |
| 11–19 mm | 10 | 15 | 5 | 7/30 (23.3%) |
| 20–29 mm | 2 | 14 | 5 | 12/21 (57%) |
| >30 mm | 1 | 8 | 1 | 6/10 (60%) |
| Node positive | 0/30 (0%) | 24/55 (43.6%) | 7/18 (38.9%) | 31/103 (30.1%) |

At all size ranges, cancers detected in the FIS were associated with a higher incidence of nodal metastases ($\chi^2=7.1$, $P<0.01$, Figure 2). In particular, cancers ⩽10 mm were associated with nodal metastases in 4/81 (5%) of PS-detected cases compared with 6/42 (14.3%) of FIS-detected cases. The relationship between size, grade and lymph node status for FIS-detected cancers is shown in Table I. Corresponding results for PS-detected cancers have been reported previously (Walls *et al.*, 1993).

### Discussion

The United Kingdom breast screening guidelines target the detection of small breast cancers, but for screening to succeed and reduce the mortality from breast cancer lesions must be detected before the development of nodal metastases (Crisp *et al.*, 1993). Breast screening theory supposes that prevalence screens are biased towards the detection of slow-growing, less aggressive cancers, whereas incidence screens detect smaller, faster growing and biologically more aggressive lesions, because of the phenomenon of length time bias (Cole, 1980). Our results are compatible with breast screening theory.

In the two screening rounds, invasive cancers of similar histological type and almost identical grade were detected. A greater proportion of cancers detected in the FIS were ⩽10 mm in size, but despite this overall lymph node positivity was significantly higher in the FIS.

Axillary lymph nodes are the main site of regional metastases from breast cancer. Lymph node status is recognised to be the most useful marker of distant metastatic spread and prognosis in patients with breast cancer (Fisher *et al.*, 1975, 1983; Valagussa *et al.*, 1978) and is the basis for several prognostic indices (Todd *et al.*, 1987; Galea *et al.*, 1992). Despite this, the management of the axilla in patients with screen-detected and early symptomatic breast cancer is controversial and ranges from no surgical intervention, to axillary sampling of ten nodes and to formal level III axillary clearance. It is not the purpose of this paper to discuss the relative merits of each of these management options, however the quality assurance guidelines laid down for surgeons managing symptomatic breast cancer advise that axillary surgery of some type must be performed in all cases (British Association of Surgical Oncologists, 1995).

For screen-detected cancers, our unit policy is to perform a level III axillary clearance for women considered to be at significant risk of having nodal metastases (i.e. >10%). For patients with cancers at low risk (<10%) of nodal involvement, no form of axillary surgery is performed. This policy is recommended by other authors (Silverstein *et al.*, 1994; Chada *et al.*, 1994; Cady, 1994) and can be justified if this important subgroup of women can be accurately identified preoperatively.

For breast cancers that present symptomatically, axillary dissection should ideally be performed at the same time as therapeutic excision of the primary tumour, the diagnosis having been made preoperatively by fine-needle aspiration cytology. However, stereotactic cytological assessment of small impalpable screen-detected cancers can be difficult, and many of our patients require diagnostic localisation biopsy.

In our previous study, PS-detected cancers ⩽10 mm diameter were associated with lymph node metastases in only 5% of cases. No grade I cancers <30 mm were associated with nodal involvement. We recommend that this subgroup of patients can be spared axillary surgery after localisation biopsy. This reduces morbidity and operating time, with little loss of prognostic information.

Apart from grade I cancers, this low rate of nodal metastases was not found in cancers detected at the FIS. For any given size, cancers detected at our PS have fewer lymph node metastases than those detected at the FIS (Figure 2). Their relationship between size and lymph node status seen for PS- and FIS-detected cancers is similar to that seen between screen-detected and symptomatic cancers, with the latter having a higher incidence of nodal involvement (Crisp *et al.*, 1993). Moreover, the frequency of node metastases in the FIS-detected cancers is equivalent to that seen in a symptomatic breast cancer population (Fisher *et al.*, 1983).

The implementation of our previous recommendations led initially to fewer women with screen-detected cancer undergoing axillary surgery in Manchester, UK. In this study, patients who did not undergo axillary clearance were assumed to be lymph node negative, and we calculated lymph node positivity as a proportion of the total invasive group, and not just of those undergoing axillary dissection. If we consider axillary node involvement as a percentage of those undergoing axillary surgery, the frequency of nodal metastasis in the FIS cancer is nearly 9% higher at 38.4%.

The incidence of lymph node positivity for non-screened, symptomatic cancers has been reported to be between 40% and 50% (Crisp *et al.*, 1993; Fentiman, 1991). Therefore, in terms of their lymph node status, many of our FIS-detected cancers behave more like symptomatic than screen-detected cancers.

The two main prerequisites for a reduction in breast cancer mortality from the breast screening programme are (1) a more favourable stage distribution in screen-detected cancers, with fewer nodal metastases at presentation, and (2) a low incidence of cancers presenting with symptoms between screening rounds (interval cancers). In the North West Region, the incidence of interval cancers in the third year after breast screening approaches that which would be expected in the absence of screening (Woodman *et al.*, 1995). This suggests that a screening interval of 3 years is too long. The screening interval may have to be shortened to 2 years to reduce the incidence of interval cancers. Although no evidence as yet exists, a shortening of the screening interval may also be required to reduce the incidence of nodal metastases in FIS-detected cancers.

In conclusion, knowledge of axillary lymph node status allows accurate selection of those women requiring systemic adjuvant chemotherapy. Inadequate staging will result in a proprotion of these women receiving suboptimum therapy. It is also imperative to the monitoring of the UK screening programme, that histological evidence of node status be available on all patients at high risk of nodal metastases (Alexander *et al.*, 1994).

We believe that our recommendations for small and well-differentiated cancers detected at the PS remain valid. However, in the light of our finding of a higher incidence of node positivity in FIS-detected cancers (with the possible exception of grade I cancers), we now consider surgical staging of the axilla essential for all cancers detected at the FIS, until more reliable predictors of biological aggressiveness and cancer prognosis are identified.

### References

ALEXANDER FE, ANDERSON TJ, BROWN HK, FORREST APM, HEPBURN W, KIRKPATRICK AE, MCDONALD C, MUIR BB, PRESCOTT RJ, SHEPHERD SM, SMITH A AND WARNER J. (1994). The Edinburgh randomised trial of breast cancer screening: results after 10 years follow-up. *Br. J. Cancer*, **70**, 542–548.

ANDERSON TJ, ALEXANDER F, CHETTY U, KIRKPATRICK A, ROBERTS MM, LAMB J, LUTZ W, FORREST APM, MUIR B AND HUGGINS A. (1986). Comparative pathology of prevalent and incident cancers detected by breast screening. *Lancet*, **1**, 519–522.

ANDERSON TJ, LAMB J, DONNAN P, ALEXANDER FE, HUGGINS A, MUIR BB, KIRKPATRICK AE, CHETTY U, HEPBURN W, SMITH A, PRESCOTT RJ AND FORREST APM. (1991). Comparative pathology of breast cancer in a randomised trial of screening. *Br. J. Cancer*, **64**, 108–113.

BRITISH ASSOCIATION OF SURGICAL ONCOLOGY. (1995). Guidelines for surgeons in the management of symptomatic breast disease in the United Kingdom. *Eur. J. Surg. Oncol.*, **21**, (Suppl A).

CADY B. (1994). The need to re-examine axillary lymph node dissection in invasive breast cancer. *Cancer*, **73**, 505–508.

CHADA M, CHABON AB, FRIEDMANN P AND VIKRAM B. (1994). Predictors of axillary lymph node metastases in patients with T1 breast cancer. A multivariate anlaysis. *Cancer*, **73**, 350–353.

COLE P AND MORRISON AS. (1980). Basic issues in population screening for cancer. *J. Natl Cancer Inst.*, **65**, 1263–1272.

CRISP WJ, HIGGS MJ, COWAN WK, CUNLIFFE WJ, LISTON J, LUNT LG, PEAKMAN DJ AND YOUNG JR. (1993). Screening for breast cancer detects tumours at an earlier biological stage. *Br. J. Surg.*, **80**, 863–865.

ELSTON CW. (1987). Grading of invasive carcinoma of the breast. In *Diagnostic Histopathology of the Breast*, Page DL and Anderson TJ. (eds) pp. 300–311. Churchill Livingstone: Edinburgh.

FENTIMAN IS AND MANSEL RE. (1991). The axilla: not a no-go zone. *Lancet*, **337**, 221–223.

FISHER B, SLACK N, KATRYCH D AND WOLMARK N. (1975). Ten-year follow-up results of patients with carcinoma of the breast in a cooperative clinical trial evaluating surgical adjuvant chemotherapy. *Surg. Gynecol. Obstet.*, **140**, 528–534.

FISHER B, BAUER M, WICKERMANN L, REDMOND CK AND FISHER ER. (1983). Relation of number of positive nodes to the prognosis of patients with primary breast cancer. *Cancer*, **52**, 1551–1557.

GALEA MH, BLAMEY RW, ELLIS IO AND ELSTON CW. (1992). The Nottingham prognostic index in primary breast cancer. *Breast Cancer Res. Treat.*, **22**, 207–220.

SILVERSTEIN MJ, GIERSON ED, WAISMAN JR, SENOFSKY GM, COLBURN WJ AND GAMAGAMI P. (1994). Axillary lymph node dissection for T1a breast carcinoma. Is it indicated? *Cancer*, **73**, 664–667.

TABAR L, FABERBERG G, DUFFY SW, DAY NE, GAD NE AND GRONTOFT O. (1992). Update of the Swedish two-county program of mammographic screening for breast cancer. *Breast Imaging: Current Status and Future Directions*, **33(i)**, 187–210.

TODD JH, DOWLE C AND WILLIAMS MR. (1987). Confirmation of a prognostic index in primary breast cancer. *Br. J. Cancer*, **56**, 489–492.

VALAGUSSA P, BONADONNA G AND VERONESI U. (1978). Patterns of relapse and survival following radical mastectomy. *Cancer*, **41**, 1170–1178.

WALLS J, BOGGIS CRM, WILSON M, ASBURY D, ROBERTS JV, BUNDRED NJ AND MANSEL RE. (1993). Treatment of the axilla in patients with screen-detected breast cancer. *Br. J. Surg.*, **80**, 436–438.

WOODMAN CBJ, THRELFALL AG, BOGGIS CRM AND PRIOR P. (1995). Is the three year screeing interval too long? Occurrence of interval cancers in NHS breast screening programme's north western region. *Br. Med. J.*, **310**, 224–226.